# Complete Genome Sequence of a Variant Porcine Epidemic Diarrhea Virus Strain Isolated in Central China

**DOI:** 10.1128/genomeA.00243-12

**Published:** 2013-02-21

**Authors:** Xiao-Meng Wang, Bei-Bei Niu, He Yan, Dong-Sheng Gao, Jin-Yao Huo, Lu Chen, Hong-Tao Chang, Chuan-qing Wang, Jun Zhao

**Affiliations:** College of Animal Science and Veterinary Medicine, Henan Agricultural University, Zhengzhou, Henan Province, China

## Abstract

We report here the complete genome sequence of the porcine epidemic diarrhea virus strain CH/ZMDZY/11 isolated from central China. Our data, together with sequence data of porcine epidemic diarrhea virus (PEDV) isolates from other parts in China, will help to understand better the epidemiology and genetic diversity of PEDV field isolates in China.

## GENOME ANNOUNCEMENT

Porcine epidemic diarrhea virus (PEDV), a positive-sense, single-stranded RNA virus belonging to group 1 of the genus *Coronavirus* of the family *Coronaviridae*, is a causative of porcine epidemic diarrhea (PED), an acute and highly contagious enteric disease characterized by watery diarrhea, vomiting, dehydration, and high mortality in nursery piglets (1). Since December 2010, PED outbreaks have been observed in most of the provinces in China. To date, the complete genome sequences of several PEDV strains from southern China have been reported (2–10). To understand the diversity, as well as the epidemiological and molecular characteristics, of PEDV isolates in China, we sequenced the complete genome of CH/ZMDZY/11, a virulent PEDV strain that was isolated from a suckling piglet with severe diarrhea in Henan, a central province in China.

Based on the sequence of PEDV strain CV777, 19 pairs of oligonucleotide primers were designed to amplify the different regions of the CH/ZMDZY/11 genome. The PCR products were gel-purified and cloned into pMD18-T vector (TaKaRa Biotechnology [Dalian]) and sequenced with an Applied Biosystems (ABI) 3730xl DNA analyzer. All fragments were sequenced in both directions and confirmed. Sequence assembling and alignment were performed using the Lasergene sequence analysis software (DNAStar Inc., Madison, WI). The whole genome of the CH/ZMDZY/11 strain is 28,038 nucleotides (nt) in length, excluding the poly(A) tail. Its genome organization is similar to those of other reported PEDVs, with the typical gene order 5′-ORF1a/1b–S–ORF3–E–M–N -3′ (1, 11). The 5′ and 3′ ends of the genome contain 292-nt and 334-nt untranslated regions, respectively. The whole genome contains seven coding regions. The ORF1a/1b gene (nt 293 to 20637) encodes two large nonstructural precursor polyproteins: replicase 1a (nt 293 to 12601) and replicase 1b (nt 12601 to 20637). The S gene contains 4,161 nt (nt 20634 to 24794), ORF3 contains 675 nt (nt 24794 to 25468), the E gene contains 231 nt (nt 25449 to 25679), the M gene contains 681 nt (nt 25687 to 26368), and the N gene contains 1326 nt (nt 26379 to 27704).

Compared to other previously reported Chinese PEDV strains, the complete genome sequence of CH/ZMDZY/11 exhibited different nucleotide sequence identities: 99.1% with BJ-2011-1 and GD-B; 98.9% with CH-FIND-3-2011; 98.8% with AJ1102 and LC; 98.2% with GD-A, CH-GD-1, and ZJCZ4; and 97.3% with CH–S. The amino acid sequence of the S gene of CH/ZMDZY/11 showed 92.5% to 99.1% homology with those of other reported Chinese strains. Compared with CH/S, LZC, CV777, and attenuated and virulent DR13 (12), there is a discontinuous 5-amino-acid insertion (^59^QGVN^62^ and ^140^N), a 3-amino-acid replacement (^160^DGK^162^ to ^160^EHS^162^) and a 2-amino-acid deletion (^163^D/NI^164^) in the N-terminal region of the S gene of CH/ZMDZY/11. These insertions, replacements, and deletions were also observed in other Chinese strains isolated after 2010. There is a continuous 17-amino-acid deletion in the ORF3 gene of attenuated DR13 (^82^YCPLLYYCGALLDATII^98^). However, this deletion does not exist in CH/ZMDZY/11 and other Chinese strains.

### Nucleotide sequence accession number.

The complete genome sequence of PEDV strain CH/ZMDZY/11 was submitted to GenBank under the accession no. KC196276.
